# Efficacy of low level laser therapy on neurosensory recovery after injury to the inferior alveolar nerve

**DOI:** 10.1186/1746-160X-2-3

**Published:** 2006-02-15

**Authors:** Tuncer Ozen, Kaan Orhan, Ilker Gorur, Adnan Ozturk

**Affiliations:** 1 Gülhane Military Medical Academy, Department of Oral Diagnosis and Radiology, 06018, Etlik, Ankara, Turkey; 2 Ankara University, Faculty of Dentistry Department of Oral Diagnosis and Radiology, 06500, Besevler, Ankara, Turkey; 3 Ankara University, Faculty of Dentistry Department of Oral and Maxillofacial Surgery, 06500, Besevler, Ankara, Turkey

## Abstract

**Background:**

The most severe complication after the removal of mandibular third molars is injury to the inferior alveolar nerve or the lingual nerve. These complications are rather uncommon (0.4% to 8.4%) and most of them are transient. However, some of them persist for longer than 6 months, which can leave various degrees of long-term permanent disability. While several methods such as pharmacologic therapy, microneurosurgery, autogenous and alloplastic grafting can be used for the treatment of long-standing sensory aberrations in the inferior alveolar nerve, there are few reports regarding low level laser treatment. This paper reports the effects of low level laser therapy in 4 patients with longstanding sensory nerve impairment following mandibular third molar surgery.

**Methods:**

Four female patients had complaints of paresthesia and dysesthesia of the lip, chin and gingiva, and buccal regions. Each patient had undergone mandibular third molar surgery at least 1 year before. All patients were treated with low level laser therapy. Clinical neurosensory tests (the brush stroke directional discrimination test, 2-point discrimination test, and a subjective assessment of neurosensory function using a visual analog scale) were used before and after treatment, and the responses were plotted over time.

**Results:**

When the neurosensory assessment scores after treatment with LLL therapy were compared with the baseline values prior to treatment, there was a significant acceleration in the time course, as well as in the magnitude, of neurosensory return. The VAS analysis revealed progressive improvement over time.

**Conclusion:**

Low level laser therapy seemed to be conducive to the reduction of long-standing sensory nerve impairment following third molar surgery. Further studies are worthwhile regarding the clinical application of this treatment modality.

## Background

The close anatomic relationship between the inferior alveolar nerve (IAN) and the roots of an impacted mandibular third molar tooth is well known. Therefore, the possibility of injury to the IAN resulting in paresthesia in the course of the surgical removal of the impacted mandibular third molars has been widely demonstrated [1-5]. The incidence of nerve damage after the removal of mandibular third molar teeth ranges from 0.4% to 8.4% [6-13]. In the majority of cases, altered sensation is a transitory phenomenon [1,15]. However, some persist for longer than 6 months, which can leave various degrees of long-term permanent disability.

Presently, there is no standardized protocol in the evaluation and management of patients with IAN injury. There are several methods which can be used for the treatment of longstanding sensory aberrations in the IAN. A multitude of surgical modalities are currently used in nerve repair including epineural repair, perineural repair, autogenous interpositional nerve grafts, vein grafts, and entubulation, with or without the use of neurotrophic and neurotropic factors which apply to the IAN [6,16-20]. Some persistent nerve alterations may be due to scar tissue entrapment of the nerve causing a conduction block or preventing regeneration as a result of compression. This generally requires an external or internal neurolysis [6,15]. Anatomic and physiologic studies have confirmed that injured nerve trunks might form neuromas [21]. In the case of neuroma formation, the resection of the neuroma and debridement of the nerve segments is undertaken until healthy neural fascicles are encountered [6]. When neural tissue is resected or has been previously destroyed, a gap forms between proximal and distal nerve stumps. Direct approximation of the nerve stumps would result in harmful tension across the suture line. Either the nerve should be mobilized, rerouted or a nerve graft interposed to avoid longitudinal suture line tension [6,35]. If primary anastomosis can not be achieved without tension, then a sural, greater auricular or medial ante-brachial cutaneous nerve may be necessary to span a large nerve defect. However, this requires a second surgical site with increased morbidity at the graft harvest site [6,36,37].

The long term results of surgical procedures which appear to be varied and anecdotal, have been inconclusive in the current and past literature [14,20]. Some studies state that early nerve repair appears to provide better results than does late repair [20,38,39]. However, this is not universally accepted. There are other reports in the literature which state that timing has little effect on the success of nerve repair procedures [40,41]. There is no consensus on exactly what constitutes an early versus late repair, because some have advocated that late repairs are those performed after 1 year and others have stated that they are repairs performed after 3 months [14]. Another critical issue is performing the indicated surgery. It seems that direct nerve anastomosis provides better results than a graft. Meanwhile, the data from recent preliminary studies also shows that the use of Gore-Tex tubes as grafts may be unsuccessful [42,43]. Rutner et al [20] concluded that trigeminal microsurgery is indicated in patients with a peripheral neuropathic lesion and clinically accompanied by hyperalgesia and hyperpathia. Patients with dysesthesia had more neuroses and depression with long-standing pain symptoms which have a poor prognosis with microsurgery.

It is apparent from the literature that the value of surgical approaches to the IAN which have been described for the management of IAN injury remain uncertain, and indeed some procedures may do harm than good [16].

Besides these surgical modalities, low level laser (LLL) therapy can also used for the treatment of nerve injuries. There have been many claims for the therapeutic effects of LLL treatment such as acceleration of wound healing [22], pain attenuation [4,24,23], restoration of normal neural function following injury [6,11,25,26], enhanced remodeling and repair of bone, normalization of abnormal hormonal function, stimulation of endorphin release and modulation of the immune system. Published data on efficacy exist for some but not all of these applications [27]. There are several studies reported in the treatment of IAN injury. These studies were reported by Midamda [28], and Khullar et al. [11,44] in both subjective and objective neurosensory impairment after LLL treatment of IAN paresthesia. Recently, Miloro et al reported the LLL effect both on neural regeneration in surgically created defects [6], and on neurosensory recovery after sagittal ramus osteotomy [25]. They used gallium-aluminum-arsenide (GaAlAs) LLL in patients with neurosensory impairment, and they reported both subjective and objective improvement in IAN after LLL treatment. Because LLL is relatively noninvasive, its ability to stimulate injured nerves without surgical intervention is desirable [36]. There have been only a few studies recently reported in the literature about the influence of LLL on neural regeneration especially IAN. Hence, it was considered worthwhile to see the effects of low-level laser treatment with GaAlAs laser resulting in objectively and subjectively verified improvement in sensory perception after a long-standing post-surgical inferior alveolar nerve injury, and to make a contribution to the studies in the literature about this treatment modality.

## Methods

The subjects consisted of 4 female patients with an age range of 21–24 years with post-surgical sensory abnormalities lasting longer than 1 year in the distribution of the inferior alveolar nerve subsequent to surgical removal of impacted mandibular third molar teeth (Table 1). Surgeons who were graduate dentists specializing in Oral Surgery (in their second, or third year) performed all the extractions employing a common surgical procedure. The surgical field and all surgical materials were sterile. All subjects received IAN block injections of 1.8 ml of 2% lidocaine (36 mg) with 1:100,000 epinephrine (18 μg), (Xylocaine, Dentsply Pharmaceutical, York, PA). The Inferior alveolar nerve anesthetic technique was used (direct truncal block) for the IAN block. Infiltrating anesthesia was also performed in the vestibular region innervated by the buccal nerve to ensure that the surgical fields were fully anesthetized. Submucosal injection was also made in the vestibular fundus of the region of the lower and third molar using a standard 27-gauge 1 ^1/2 ^inch (Monoject, Sherwood Medical, St. Louis, MO) needle attached to a standard aspirating syringe. After the target area was reached and aspiration performed, the anesthetic solution was deposited over a 1 minute time period. A standard incision was used from the anterior border of the ramus to the disto-facial corner of the second molar following the buccal gingival sulcus along the second and first molars. After periosteal elevation, the bone surrounding the third molar was removed with a round bur in a handpiece using a copious amount of saline irrigation. The third molars were split using a tungsten fissure bur and a straight elevator as the routine technique. The tooth was then removed in several pieces. The alveolus was inspected for granulation tissue followed by copious irrigation with saline. Closure was accomplished with 3-0 silk sutures. A gauze pack was pressed against the surgical site and the patient was instructed to bite upon this for half an hour. Following the surgery, the patient was given no medication other than post-operative antibiotics and analgesics.

**Table 1:** The details of the patients with neurosensory deficit after third molar surgery

Patient	Date of Surgery (mo,yr)	Age at start of treatment (yrs)	Time after injury before starting treatment (months)
I	11,2004	21	15
II	6,2003	24	21
III	5,2004	24	12
IV	1,2004	22	18

**Figure 1:**
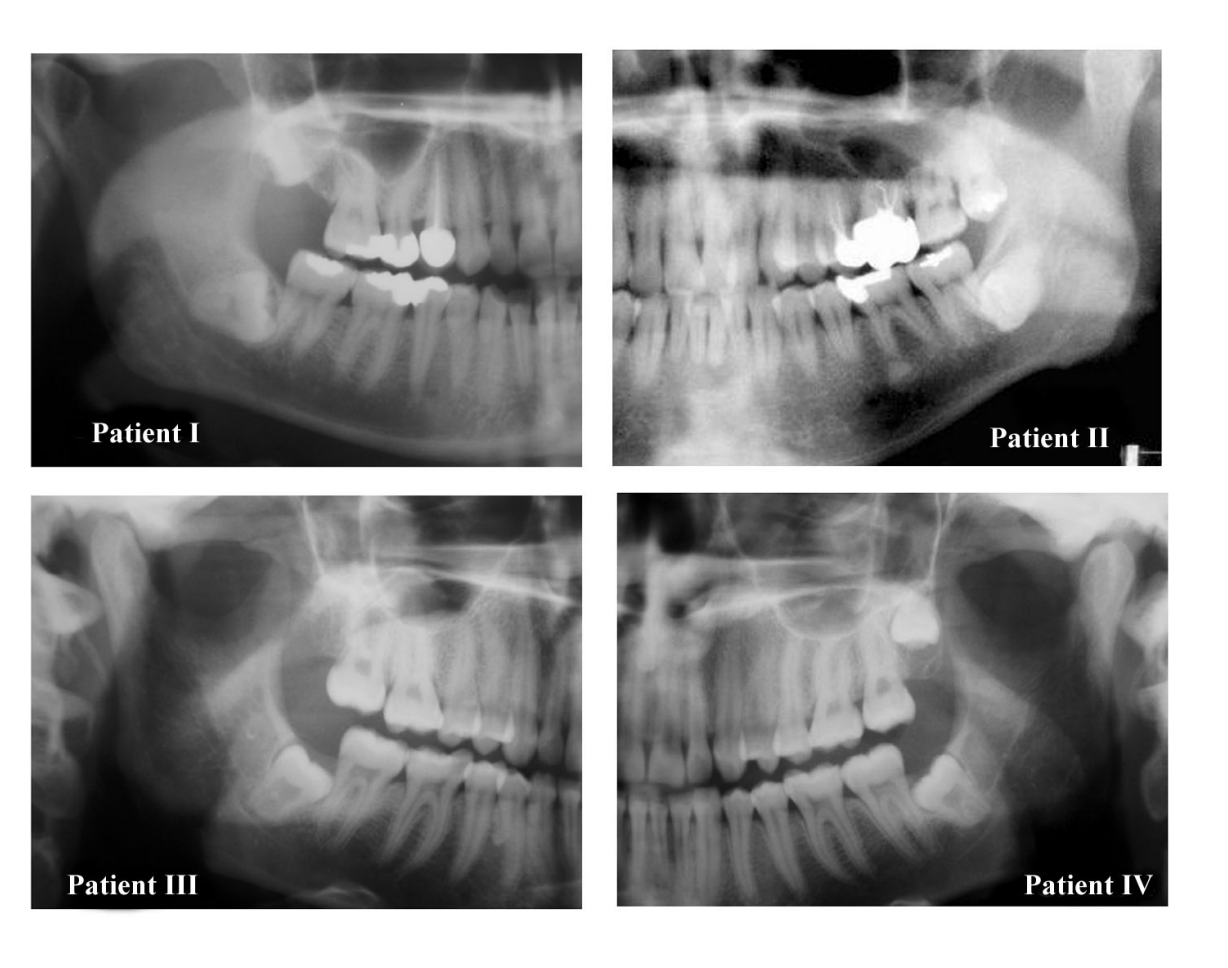
A montage of the panoramic radiographs of the four patients showing in each case the close spatial relationship between the mandibular third molar teeth and the IAN.

After the third molar surgeries, subsequent neurosensory impairment had occurred in the patients. These impairments were as follows: two patients had slightly painful dysesthesia of lip, chin and gingival regions (patients II and IV), while patient I had hypoesthesia on the area of the chin, gingiva, buccal regions and the lips, and patient III had complete paresthesia caused by the injury of the IAN. Besides these impairments, the clinical examination of the patients also revealed no alterations of sensation in the tongue, no taste problems or thermal sensation problems. The pre-operative panoramic radiographs revealed the close relationship between the right mandibular third molar tooth and the IAN before surgery (Figure 1). It was thought that these impairments had formed due to either the third molar surgery or the local anesthetic injection with or without direct needle trauma. No treatment, surgical or otherwise, had been provided for the treatment of these complaints after surgery. According to Tay [7], the persistence of sensory alteration at 1 year suggests the presence of Sunderland fourth-degree injury. It was indicated in the surgical records of the patients that there were no complete transections. Thus, using Sunderland's classification in accordance with several studies [6,45], these patients were classified as at best third-degree with intraneural fascicular disruption and/or scarring.

All patients were reviewed on the first postoperative day and again 1 week after surgery. These patients were also monitored after 15 days and 1, 3, 6 and 9 months for recovery. After the 9-month follow-up, patients I and III could not be reached until they referred back to our clinic. The other patients (patients II and IV) were monitored at the 1-year mark and also at 18 months. After 18 months, the injury was considered to be permanent for these patients. Overall, the mean of the follow-up period of the patients was 13.5 months with a range of 9 to 18 months. All examinations and treatments were performed with the signed consent of the patient as well as the presence of a witness. All four patients decided to undergo treatment with LLL.

The study was conducted on a double blind basis. The treatments took place over a time period of 39 days at 2 day intervals following the protocol of Khullar et al [11]. The LLL treatments and recording of data were performed by a second doctor not involved in any of the surgeries, and the analysis of the recorded data was performed by a third doctor.

### LLL treatment

**Figure 2:**
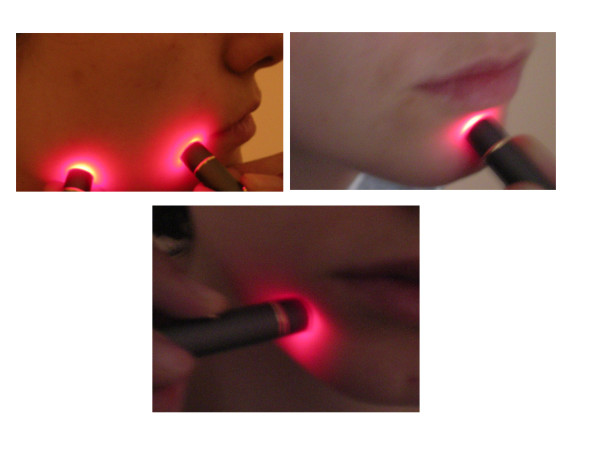
The extra-oral LLL treatment points used.

**Figure 3:**
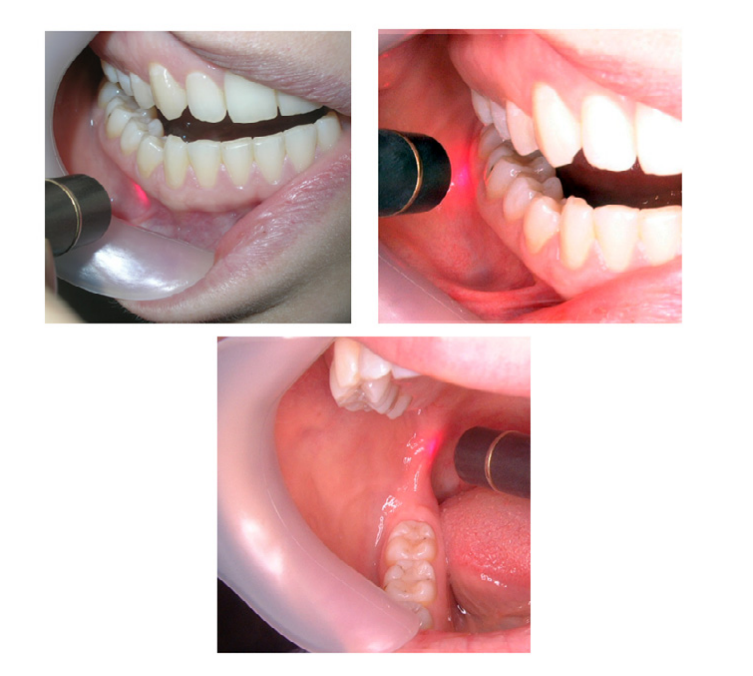
The intra-oral LLL treatment points used.

A photon-plus GaAlAs diode laser LLL system (Laser Medical Systems, ApS, Hedehusene, Denmark) was used. The unit had a contact probe with a laser beam diameter of 0.5 cm. The system delivers a 70 mW output that emits a wavelength of 820-to-830 nm. The irradiance used was 6.0 J per treatment site, which was delivered by applying 5 mW in continuous wave mode for approximately 90 seconds. Each patient received a total of 20 LLL treatment sessions. The patients were treated at 2 day intervals, 3 times a week until all sessions were completed. The laser probe was applied directly to the treatment sites. The patients experienced no sensation when the laser treatments were being carried out. A beeping noise was made at the beginning and at the end the end of the treatment. The treatment time per point was 90 seconds. Thus, one treatment session, consisting of 5 treatment sites, took approximately 8 minutes. The treatment sites were as follows: extraorally: the lower lip, chin and the region of mental foramen (Figure 2); intraorally: the mental foramen region, buccally in the region of the apicies of the first molar, and lingually in the region of the mandibular foramen (Figure 3).

### Assessment of neurosensory deficit

To determine the degree of sensory nerve damage, both objective and subjective measurements were made. All patients underwent a complete pre- and post-treatment clinical neurosensory test (CNT), as described previously by Pogrel [14] and Miloro et al [25]. This consisted of three parts: a brush stroke directional discrimination test for fine touch and direction sense, a 2-point discrimination test, and subjective assessment of the neurosensory deficit using a visual analog scale (VAS). All tests were performed in a dark, quiet room, with the patient's eyes closed. The neurosensory tests were explained to the patient and performed on a control site (i.e., hand, or arm) to confirm that the patient understood the test before formal testing of the IAN. The brush stroke directional discrimination test was performed first. A fine No.2 sable brush that consistently evoked sensation in the injured area was chosen. This was then used to map the area of paresthesia, and the brush rubbed across the test area in an anterior or posterior direction. The patient's responses to identifying the direction of movement were recorded as the number correct out of 10 tests. For the second test, 2-point discrimination was performed by using a Boley gauge with blunt points, which was intended to elicit a non-painful response. The number of millimeters of separation that could be discerned consistently was used as the discrimination value for this test. Subjective neurosensory assessment was performed by using a 10-cm, 5-degree VAS with divisions at 2.5 cm intervals as described previously by Miloro et al [25]. The divisions on the VAS scale were as follows; 1- Complete absence of sensation 2- Almost no sensation 3- Reduced sensation 4- Almost normal sensation 5- Fully normal sensation. Patients were asked to make an "x" on the line at each testing session. The distances along the line were measured and recorded. Although the study group was quite small, the Wilcoxon statistical test was performed using the SPSS 11.0 package (SPSS Inc., Chicago, IL) for Windows (*p *<*0.05*) for the evaluation of statistical significance.

## Results

**Figure 4:**
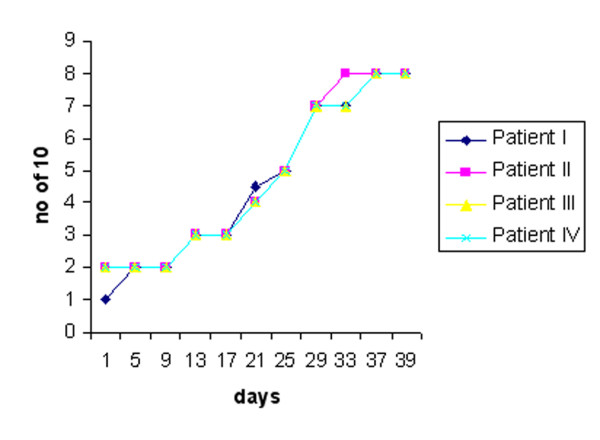
Mean results for the brush stroke directional discrimination test, with the vertical axis representing number of scores correct out of 10 (0 = indicating the pretreatment, and the x-axis indicating the days during LLL treatment).

**Figure 5:**
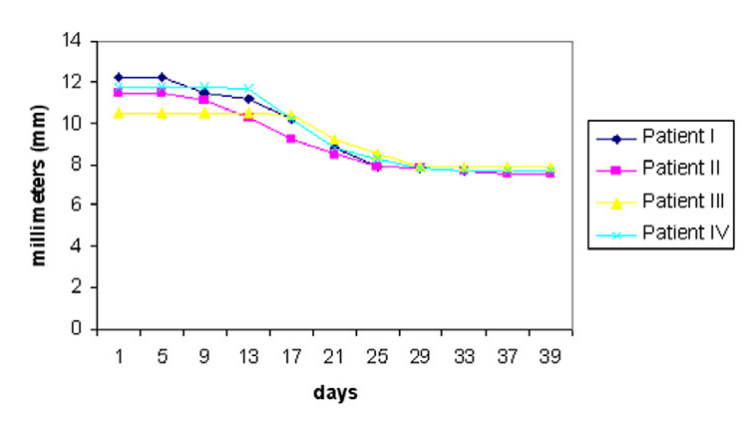
Mean results for the 2-point discrimination test, with the data expressed in millimeters.

All patients fully cooperated as regards their treatment sessions and all completed the treatment sessions. The patients reported no side effects during or after the LLL treatment. The average and standard deviations of the patients before LLL treatment were; 1.75 (s.d. 0.5) (number correct out of 10 tests for brush stroke directional discrimination test in Figure 4), 11.49 (s.d.0.73) millimeters (for 2-point discrimination test in Figure 5). After LLL treatment, it was constituted as; 8 for brush stroke test, and 7.7 (s.d.0.16) for 2-point discrimination test. Statistically, when subjective assessment was evaluated, there was a significant improvement in the assessment of the degree of neurosensory deficit (*p *= *0,02*), while there were also statistical significant improvement in the brush stroke directional discrimination test for fine touch and direction sense (*p *= *0.01*). Although there is a tendency toward improvement in the 2-point discrimination test, no statistical difference can be found between the pre and post treatment values (*P *= *0.07*).

**Figure 6:**
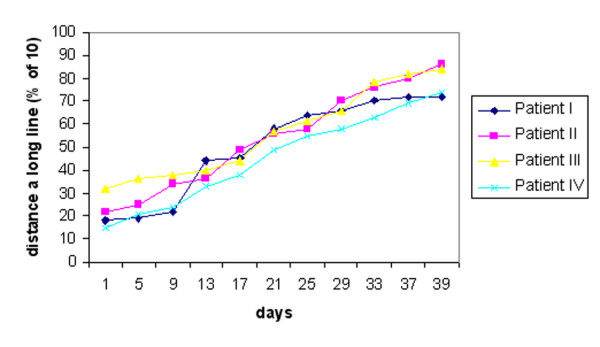
VAS scores over time, expressed as a percentage of full sensation.

In general, when the neurosensory assessment scores after treatment with LLL therapy were compared with the baseline values prior to treatment, there was progressive improvement over time, signifying return of neurosensory functions. In all patients the responses to identifying the direction of movement were increased dramatically. Before the LLL treatment, the patients average responses (Figure 4). This was accompanied by a significant improvement in the 2-point discrimination test in all patients (Figure 5). Subjective assessment using the VAS also showed an improvement over time (Figure 6). Thus, in all patients, sensation alterations were changed in a positive manner as assessed both subjectively and objectively.

## Discussion

Involvement of, and damage to, the inferior alveolar may result from a variety of clinical circumstances. Maxillofacial trauma or involvement by neoplastic growth can cause sensory dysfunction, but many cases occur as a result of dental treatment [15,46]. The most common cause is dentoalveolar surgery, in particular, the removal of mandibular third molars [5-7,13,14]. Other causes include orthognatic surgery [21,25,44,47], surgery for the management of pathologic lesions of the jaws (most commonly, cystic lesions) [48], root canal therapy [49,50], implant treatment [35,51], and injection of a local anesthetic nerve block [33,52-55]. Although most cases of nerve damage are transient and spontaneously resolve uneventfully with minimal sequelae, some persist [7]. There is generally little improvement in sensation on the injured side when assessed either by objective means or subjectively by the patient 9 months after compression injury and 12 months after nerve section [11,56].

Several classification systems have been proposed for nerve injuries, the best known being those by Seddon [31] and Sunderland [32]. Seddon classified nerve injuries into four classes: neuropraxia-conduction block resulting from mild trauma, without axonal damage; axonotmesis-more severe injury, with preservation of nerve sheath; neurotmesis-most severe injury- with nerve severance and anesthesia in the nerve distribution. The Sunderland classification expands the Seddon classification of neuropraxia, axonotmesis, and neurotmesis into fifth-degree nerve injury in increasing order of severity [6,33]. In Sunderland's second-, third, and fourth degree injuries, the afferent or efferent fibers are damaged, but the endoneurium and perineurium remain intact. Fifth-degree injury implies nerve transsection. In our cases, no surgical attempt was performed for treatment; thus, it was impossible to classify these patients precisely. However, in our opinion the persistence of sensory alteration at 1 year suggests that the patients in this study have at best a Sunderland third-degree injury, or in fact, are more close to having a fourth-degree injury.

Possible mechanisms of nerve injury in patients who sustain sensory deficits after third molar surgery with observed, intact IAN bundles include compression injury or crush injury. The process of nerve regeneration after compression or less severe crush injuries usually requires several weeks to 6 months. If there is no sensory recovery during this time, permanent loss of continuity in the nerve trunk should be expected [7]. Mandibular third molars, which have been radiographically judged to be in close proximity to the mandibular canal, have in several studies been linked to an elevated risk for postoperative complications such as nerve damage [1-4]. Besides this, several studies have indicated that local anesthesia itself can also cause IAN damage, although such situations are quite rare [52-55]. In a retrospective study, Haas and Lennon [57] reported the incidence as 1:785 000 injections while in another study, the incidence is cited as between 1:67 000 and 1:200 000 [5]. Also, studies conclude that IAN injury occurs more frequently in later years [[5,9,12], and [58]]. Bataineh [13] figured out a statistical significance between the experience of operator and IAN paresthesia.

In this study, the subjects were young female individuals and pre-operative radiographs demonstrated the close relationship between the mandibular canal and the third molars. The operations were performed by inexperienced graduate dentists specializing in Oral Surgery. In our opinion, the possible mechanism for nerve injury in our case series may have resulted from compression, stretching or partial section of the nerve, caused by bone fragments or iatrogenic damage to instrumentation. Other possible reason could be local anesthesia, including mechanical damage of the nerve shaft by a barbed needle, mechanical compression caused by internal hemorrhage or forced injection of the anesthetic solution, or chemical action of the anesthetic or of contaminating substances.

According to several studies, females were at higher risk of developing postoperative complications than men, and mainly women and older persons have the most severe discomfort after oral nerve damage [58,59]. In a recent report by Pogrel and Thamby, more females than males were affected with neuropathic pain [53]. Moreover, other studies which were conducted both in humans and animals indicated that females seem to have more postoperative neurosensory deficit disturbances (especially bilateral sagittal split osteotomy in humans) [20,47,60-64]. In our study, all patients who underwent LLL treatment were females. This finding is consistent with the previously published studies. Thus, it can be stated that females are less likely to undergo spontaneous recovery following nerve injury than are male subjects.

LLL therapy has been shown to both reduce the production of inflammatory mediators of the arachidonic acid family from injured nerves, and to promote regeneration following injury [27]. The therapeutic effects of LLL treatment include: acceleration of wound healing [22], pain attenuation [23,24,34], restoration of normal neural function following injury [6,11,25,26], enhanced remodeling and repair of bone, normalization of abnormal hormonal function, stimulation of endorphin release, and modulation of the immune system [27]. Clinical studies of the effects of LLL therapy on injured nerves have revealed an increase in nerve function and improved capacity for myelin production [6]. LLL treatment has been shown to be effective for promoting axonal growth in injured nerves in animal model [27,29,30]. The direct application of this technique to dentistry has yielded positive results in promoting the regeneration of IAN. Sensory aberrations following IAN injuries can have a significant impact on the quality of life. For this reason, interest has focused on the use of LLL therapy for the treatment of persistent IAN injury. Extensive use of LLL therapy in the perioral region after nerve trauma has been investigated by several researchers [6,11,25,28,44]

In a previous blind clinical trial Khullar et al. [11] investigated the effects of LLL treatment using a GaAlAs laser on sensory perception in a 15 patient population after a long-standing post surgical IAN injury. The average time after injury before starting treatment was 33.4 months. Six patients received real LLL treatment while the seven received placebo laser treatment. The results demonstrated an overall significant improvement in mechanosensory perception subsequent to laser treatment compared with the placebo LLL treated group. The patient in the real laser treated group showed a 44% improvement after LLL treatment. The result of this study is supported by an animal study demonstrating the regeneration of motor nerves. In this animal study, LLL treatment enhanced the recovery in terms of both the motor and sensory functions after a crush injury to the sciatic nerve [65]. Midamba and Haanaes [28] have also reported subjective improvement in patients suffering suffering from IAN injuries for more than 6 months. IAN or lingual nerve paresthesias after LLL treatment. An average subjective improvement was found (71.1%) after 20 LLL GaAlAs treatments. A further double blind study by Khullar et al [44] has reported the effect of LLL treatment on neurosensory deficit subsequent to sagittal split ramus osteotomy. They divided their study group into two groups; one (eight subjects) group received real LLL treatment, the other group received an equivalent placebo treatment. Subsequent to the completion of the 20 treatments, the real LLL treated group showed a subjective significant improvement in both lip (*p *= *0.01*) and chin (*p *= *0.02*). In addition, this group showed a significant decrease in the area of mechanoperception neurosensory deficit (*p *= *0.01*). No significant improvement was seen in the placebo group. More recently, Miloro et al. reported a positive LLL effect both on neural regeneration in surgically created defects [6], and on neurosensory recovery after sagittal ramus osteotomy [25]. They found significant improvement in neurosensory recovery after a bilateral sagittal split osteotomy, and also noted that LLL therapy may be a useful adjunct to promote neural wound healing in surgically created defects. The results in their study also showed that there was a significant improvement at 14 days and almost normal values by 2 months in level B testing, while the results of level A testing approached normal values by 14 days and virtually 100% recovery at 2 months. The results of our study are higher than those in previous studies, which do not show a 100% improvement in patients with trigeminal injuries than one year. In our opinion, this result may be due to a bias in this study. Although it appeared subjectively that all the patients were honest in their responses, the doctor who performed the tests may have had some influence on the answers. Certainly, there is significant correlation of subjective outcomes. Also in this study, there is an interesting finding: The patient with the oldest IAN injury (21 months) responded the most rapidly of all the patients. Brugnera et al. [66] treated two groups of patients with lesions to the inferior alveolar and mental nerves with LLL in their study. All cases of paresthesia were due to surgical interventions. The first group was identified as immediate and was treated within 2–15 days after the injury, while the history of injury for the second group was 30–365 days. In the first group 72.7% achieved absolute recovery and 18.3% showed relative improvement, whereas the improvement for the latter group was only 27.7%. In contrast to this report, patient II in our study made the most significant improvement in comparison to others. This finding may be consistent with a hypothesis that following damage to the nerves, the afferent and special sensory fibers have different patterns of wound healing and axonal regeneration, and that these patterns vary from person to person [61]. However, in our opinion, further studies must be conducted on this issue in order to clarify this finding.

## Conclusion

The results of the current study support the findings of the previous ones which concluded that LLL treatment results in both subjective and objective improvement in long-standing neurosensory deficit. Although several studies state that microsurgical repair of the nerve injuries can provide moderate to significant clinical neurosensory improvement after surgery, LLL therapy appears to be more beneficial and advantageous as it is non-invasive when reducing longstanding sensory nerve impairment following third molar surgery.

There is an urgent need for more studies to be undertaken and for the results of these to be disseminated widely to clinicians using LLL treatment. Only then will the aura of controversy and the stigma be removed from this area of clinical practice [27].

## Competing interests

The author(s) declare that they have no competing interests.

## Authors' contributions

KO drafted the manuscript. TO, KO, IG and AO participated in the writing of the manuscript. KO, IG and AO carried out the literature search. IG performed the treatment of the patients while KO made the pre and post-treatment clinical neurosensory tests. All authors have given the final approval of the version to be submitted to the journal. Each author has participated sufficiently in the work to take public responsibility for portions of the content.
